# Genetic Algorithm for Traveling Salesman Problem with Modified Cycle Crossover Operator

**DOI:** 10.1155/2017/7430125

**Published:** 2017-10-25

**Authors:** Abid Hussain, Yousaf Shad Muhammad, M. Nauman Sajid, Ijaz Hussain, Alaa Mohamd Shoukry, Showkat Gani

**Affiliations:** ^1^Department of Statistics, Quaid-i-Azam University, Islamabad, Pakistan; ^2^Department of Computer Science, Foundation University, Islamabad, Pakistan; ^3^Arriyadh Community College, King Saud University, Riyadh, Saudi Arabia; ^4^KSA Workers University, El Mansoura, Egypt; ^5^College of Business Administration, King Saud University, Muzahimiyah, Saudi Arabia

## Abstract

Genetic algorithms are evolutionary techniques used for optimization purposes according to survival of the fittest idea. These methods do not ensure optimal solutions; however, they give good approximation usually in time. The genetic algorithms are useful for NP-hard problems, especially the traveling salesman problem. The genetic algorithm depends on selection criteria, crossover, and mutation operators. To tackle the traveling salesman problem using genetic algorithms, there are various representations such as binary, path, adjacency, ordinal, and matrix representations. In this article, we propose a new crossover operator for traveling salesman problem to minimize the total distance. This approach has been linked with path representation, which is the most natural way to represent a legal tour. Computational results are also reported with some traditional path representation methods like partially mapped and order crossovers along with new cycle crossover operator for some benchmark TSPLIB instances and found improvements.

## 1. Introduction

Genetic algorithms (GAs) are derivative-free stochastic approach based on biological evolutionary processes proposed by Holland [1]. In nature, the most suitable individuals are likely to survive and mate; therefore, the next generation should be healthier and fitter than previous one. A lot of work and applications have been done about GAs in a frequently cited book by Golberg [2]. GAs work with population of chromosomes that are represented by some underlying parameters set codes.

The traveling salesman problem (TSP) is one of the most famous benchmarks, significant, historic, and very hard combinatorial optimization problem. TSP was documented by Euler in 1759, whose interest was in solving the knight's tour problem [3]. It is the fundamental problem in the fields of computer science, engineering, operations research, discrete mathematics, graph theory, and so forth. TSP can be described as the minimization of the total distance traveled by touring all cities exactly once and return to depot city. The traveling salesman problems (TSPs) are classified into two groups on the basis of the structure of the distance matrix as symmetric and asymmetric. The TSP is symmetric if *c*_*ij*_ = *c*_*ji*_, ∀*i*, *j*, where *i* and *j* represent the row and column of a distance (cost) matrix, respectively, otherwise asymmetric. For *n* cities, there are (*n* − 1)! possible ways to find the tour after fixing the starting city for asymmetric and its half for symmetric TSP. If we have only 10 cities, then there are 362,880 and 181,440 ways for asymmetric and symmetric TSP, respectively. This is the reason to say TSP is NP-hard problem. TSP has many applications such as variety of routing and scheduling problems, computer wiring, and movement of people, X-ray crystallography [4], and automatic drilling of printed circuit boards and threading of scan cells in a testable Very-Large-Scale-Integrated (VLSI) circuits [5].

Over the last three decades, TSP received considerable attention and various approaches are proposed to solve the problem, such as branch and bound [6], cutting planes [7], 2-opt [8], particle swarm [9], simulated annealing [10], ant colony [11, 12], neural network [13], tabu search [14], and genetic algorithms [3, 15–17]. Some of these methods are exact, while others are heuristic algorithms. A comprehensive study about GAs approaches are successfully applied to the TSP [18]. A survey of GAs approaches for TSP was presented by Potvin [17]. A new sequential constructive crossover generates high quality solution to the TSP by Ahmed [19]. A new genetic algorithm for asymmetric TSP is proposed by Nagata and Soler [20]. Three new variations for order crossover are presented with improvements by Deep and Adane [21]. Ghadle and Muley presented modified one's algorithm with MATLAB programming to solve TSP [22]. Piwonska associated a profit based genetic algorithm with TSP and obtained good results to be tested on networks of cities in some voivodeships of Poland [23]. Kumar et al. presented the comparative analysis of different crossover operators for TSP and showed partially mapped crossover gives shortest path [24]. A simple and pure genetic algorithm can be defined in the following steps.

**Step 1 .**

Create an initial population of P chromosomes.

**Step 2 .**

Evaluate the fitness of each chromosome.

**Step 3 .**

Choose P/2 parents from the current population via proportional selection.

**Step 4 .**

Randomly select two parents to create offspring using crossover operator.

**Step 5 .**

Apply mutation operators for minor changes in the results.

**Step 6 .**

Repeat Steps  4 and 5 until all parents are selected and mated.

**Step 7 .**

Replace old population of chromosomes with new one.

**Step 8 .**

Evaluate the fitness of each chromosome in the new population.

**Step 9 .**

Terminate if the number of generations meets some upper bound; otherwise go to Step  3.

The selection criteria, crossover, and mutation are major operators, but crossover plays a vital role in GAs. A lot of crossover operators have been proposed in literature and all have their significant importance. In this article, we also proposed a new crossover operator for TSP which is moved within two selected parents as previous cycle crossover operator. In Section 2, we present crossover operators for TSP and proposed a new crossover operator for path representation in Section 3; computational experiments and discussion are in Section 4 and conclusion is in Section 5.

## 2. Crossover Operators for TSP

In literature, there are many representations to solve the TSP using the GAs. Among these binary, path, adjacency, ordinal, and matrix representations are important. The further types of these representations are given in Table 1. We are limiting our self only with the path representation which is most natural and legal way to represent a tour and skip the others representations.

### 2.1. Path Representation

The most natural way to present a legal tour is probably by using path representation. For example, a tour 1 → 4 → 8 → 2 → 5 → 3 → 6 → 7 can be represented simply as (1 4 8 2 5 3 6 7).

Since the TSPs in combinatorial with the path representation and the classical crossover operators such as one-point, two-point, and uniform crossovers are not suitable, we choose only partially mapped, order, and cycle crossover operators from path representation which are mostly used in literature and also we can compare our proposed crossover operator with these operators.

#### 2.1.1. Partially Mapped Crossover Operator

The partially mapped crossover (PMX) was proposed by Goldberg and Lingle [25]. After choosing two random cut points on parents to build offspring, the portion between cut points, one parent's string is mapped onto the other parent's string and the remaining information is exchanged. Consider, for example, the two parents tours with randomly one cut point between 3rd and 4th bits and other cut point between 6th and 7th bits are as follows (the two cut points marked with “∣”):

(1) P1=3 4 8 ∣ 271 ∣ 6 5,P2=4 2 5 ∣ 168 ∣ 3 7.

The mapping sections are between the cut points. In this example, the mapping systems are 2↔1, 7↔6, and 1↔8. Now two mapping sections are copied with each other to make offspring as follows:

(2) O1=× × × ∣ 168 ∣ × ×,O2=× × × ∣ 271 ∣ × ×.

Then we can fill further bits (from the original parents), for those which have no conflict as follows:

(3) O1=3 4 × ∣ 168 ∣ × 5,O2=4 × 5 ∣ 271 ∣ 3 ×.

Hence, the first × in the first offspring is 8 which comes from first parent but 8 is already in this offspring, so we check mapping 1↔8 and see again 1 existing in this offspring, again check mapping 2↔1, so 2 occupies at first ×. Similarly, the second × in first offspring is 6 which comes from first parent but 6 exists in this offspring; we check mapping 7↔6 as well, so 7 occupies at second ×. Thus the offspring 1 is

(4) $$\begin{array}{c} O_{1}=\left(\;3\;4\;2\;|\;\begin{array}{ccc} 1 & 6 & 8 \end{array}\;|\;7\;5\;\right). \end{array}$$

Analogously, we complete second offspring as well:

(5) $$\begin{array}{c} O_{2}=\left(\;4\;8\;5\;|\;\begin{array}{ccc} 2 & 7 & 1 \end{array}\;|\;3\;6\;\right). \end{array}$$

#### 2.1.2. Order Crossover Operator

The order crossover (OX) was proposed by Davis [26]. It builds offspring by choosing a subtour of a parent and preserving the relative order of bits of the other parent. Consider, for example, the two parents tours are as follows (with randomly two cut points marked by “∣”):

(6) P1=3 4 8 ∣ 271 ∣ 6 5,P2=4 2 5 ∣ 168 ∣ 3 7.

The offspring are produced in the following way. First, the bits are copied down between the cuts with similar way into the offspring, which gives

(7) O1=× × × ∣ 271 ∣ × ×,O2=× × × ∣ 168 ∣ × ×.

After this, starting from the second cut point of one parent, the bits from the other parent are copied in the same order omitting existing bits. The sequence of the bits in the second parent from the second cut point is “3 → 7 → 4 → 2 → 5 → 1 → 6 → 8.” After removal of bits 2, 7, and 1, which are already in the first offspring, the new sequence is “3 → 4 → 5 → 6 → 8.” This sequence is placed in the first offspring starting from the second cut point:

(8) $$\begin{array}{c} O_{1}=\left(\;5\;6\;8\;|\;\begin{array}{ccc} 2 & 7 & 1 \end{array}\;|\;3\;4\;\right). \end{array}$$

Analogously, we complete second offspring as well:

(9) $$\begin{array}{c} O_{2}=\left(\;4\;2\;7\;|\;\begin{array}{ccc} 1 & 6 & 8 \end{array}\;|\;5\;3\;\right). \end{array}$$

#### 2.1.3. Cycle Crossover Operator

The cycle crossover (CX) operator was first proposed by Oliver et al. [27]. Using this technique to create offspring in such a way that each bit with its position comes from one of the parents. For example, consider the tours of two parents:

(10) P1=1 2 3 4 5 6 7 8,P2=8 5 2 1 3 6 4 7.

Now it is up to us how we choose the first bit for the offspring to be either from the first or from the second parent. In our example, the first bit of the offspring has to be 1 or 8. Let us choose it be 1:

(11) $$\begin{array}{c} O_{1}=\left(\;1\;\times \;\times \;\times \;\times \;\times \;\times \;\times \;\right). \end{array}$$

Now every bit in the offspring should be taken from one of its parents with the same position, it means that further we do not have any choice, so the next bit to be considered must be bit 8, as the bit from the second parent is just below the selected bit 1. In first parent this bit is at 8th position; thus

(12) $$\begin{array}{c} O_{1}=\left(\;1\;\times \;\times \;\times \;\times \;\times \;\times \;8\;\right). \end{array}$$

This turnout implies bit 7, which is the bit of second parent just below the selected bit at 7th position in first parent. Thus

(13) $$\begin{array}{c} O_{1}=\left(\;1\;\times \;\times \;\times \;\times \;\times \;7\;8\;\right). \end{array}$$

Next, this forced us to put 4 at 4th position as

(14) $$\begin{array}{c} O_{1}=\left(\;1\;\times \;\times \;4\;\times \;\times \;7\;8\;\right). \end{array}$$

After this, 1 comes which is already in the list; thus we have completed a cycle and filling the remaining blank positions with the bits of those positions which are in second parent:

(15) $$\begin{array}{c} O_{1}=\left(\;1\;5\;2\;4\;3\;6\;7\;8\;\right). \end{array}$$

Similarly the second offspring is

(16) $$\begin{array}{c} O_{2}=\left(\;8\;2\;3\;1\;5\;6\;4\;7\;\right). \end{array}$$

But there is a drawback that sometimes this technique produces same offspring, for example, the following two parents:

(17) P1=34827165,P2=4 2 5 1 6 8 3 7.

After applying CX technique, the resultant offspring are as follows:

(18) O1=3 4 8 2 7 1 6 5,O2=4 2 5 1 6 8 3 7,

which are the exactly the same as their parents.

## 3. Proposed Crossover Operators

We are going to propose a new crossover operator which works similarly as CX, so we suggest it as CX2. At the same time it generates both offspring from parents using cycle(s) till last bit. We differentiate CX2 in the following steps.

**Step 1 .**

Choose two parents for mating.

**Step 2 .**

Select 1st bit from second parent as a 1st bit of first offspring.

**Step 3 .**

The selected bit from Step  2 would be found in first parent and pick the exact same position bit which is in second parent and that bit would be found again in the first parent and, finally, the exact same position bit which is in second parent will be selected for 1st bit of second offspring.

**Step 4 .**

The selected bit from Step  3 would be found in first parent and pick the exact same position bit which is in second parent as the next bit for first offspring. (Note: for the first offspring, we choose bits only with one move and two moves for second offspring's bits.)

**Step 5 .**

Repeat Steps  3 and 4 till 1st bit of first parent will not come in second offspring (complete a cycle) and process may be terminated.

**Step 6 .**

If some bits are left, then the same bits in first parent and in second offspring till now and vice versa are left out from both parents. For remaining bits repeat Steps  2, 3, and 4 to complete the process.

According to the previous steps, we derive two cases for CX2. First case of CX2 will be terminated within Step  5 and second will take all six steps. We provide detailed examples of both cases in next subsections.

### 3.1. CX2: Case  1

Consider the two selected parents as mentioned in Step  1:

(19) P1=34827165,P2=42516837.

Using Step  2,

(20) $$\begin{array}{c} O_{1}=\left(\begin{array}{cccccccc} 4 & \times & \times & \times & \times & \times & \times & \times \end{array}\right). \end{array}$$

As using Step  3 which selected 4 in Step  2, where 4 is found at second position in first parent and the bit at this position in second parent is 2. For searching again, 2 is at fourth position in first parent and 1 is at same position in second parent, so 1 is selected for second offspring as follows:

(21) $$\begin{array}{c} O_{2}=\left(\begin{array}{cccccccc} 1 & \times & \times & \times & \times & \times & \times & \times \end{array}\right). \end{array}$$

To follow Step  4, the previous bit was 1 and it is located at 6th position in first parent and at this position bit is 8 in second parent, so

(22) $$\begin{array}{c} O_{1}=\left(\begin{array}{cccccccc} 4 & 8 & \times & \times & \times & \times & \times & \times \end{array}\right). \end{array}$$

And for two moves below 8 is 5 and below 5 is 7, so

(23) $$\begin{array}{c} O_{2}=\left(\begin{array}{cccccccc} 1 & 7 & \times & \times & \times & \times & \times & \times \end{array}\right). \end{array}$$

Hence similarly,

(24) O1=48625317,O2=17486253.

We see that the last bit of second offspring is 3 which was the 1st bit of first parent. Hence proposed scheme is over within one cycle.

### 3.2. CX2: Case  2

Consider the two selected parents as mentioned in Step  1:

(25) P1=12345678,P2=27584163.

Now using Step  2 of the scheme,

(26) $$\begin{array}{c} O_{1}=\left(\begin{array}{cccccccc} 2 & \times & \times & \times & \times & \times & \times & \times \end{array}\right). \end{array}$$

After this, Step  3 calls us to locate the position of bit 2 in first parent, which is at 2nd and 7 is at same position in second parent, again searching bit 7 in first parent and located at 7th position and 6 is at the same position in second parent, so we choose bit 6 for second offspring:

(27) $$\begin{array}{c} O_{2}=\left(\begin{array}{cccccccc} 6 & \times & \times & \times & \times & \times & \times & \times \end{array}\right). \end{array}$$

Continue Steps  4 and 5 as well:

(28) O1=2167××××,O2=6721××××.

The Step  5 is finished because bit 1 has come in second offspring which was in 1st position of first parent. Now before applying Step  6, we match first offspring's bits with second parent or vice versa and leave out the existing bits with their position in both parents as follwos:

(29) P1=••345••8,P2=••584••3.

Now filled positions of parents and “×” positions of offspring are considered 1st, 2nd, and 3rd positions, and so forth, so we can complete Step  6 as well:

(30) O1=2 1 6 7 ∣ 5 3 8 4,O2=6 7 2 1 ∣ 8 4 5 3.

Hence the scheme is over with efficient work.

To apply this crossover operator, we made a MATLAB code for genetic algorithms and have given pseudo-code in Algorithm 1.

## 4. Computational Experiments and Discussion

We use genetic algorithm in MATLAB software to compare the proposed crossover operator with some traditional path representation crossover operators. Our first experiment has 7 cities and we impose the transition distance between cities in Table 2. To solve this problem using GAs, the genetic parameters are set as population size, *M* = 30; maximum generation, *G* = 10; crossover probability, *P*_*c*_ = 0.8; mutation probability, *P*_*m*_ = 0.1. In this experiment, the optimal path and optimal value are 6 → 1 → 5 → 3 → 4 → 2 → 7 and 159, respectively.

Table 3 summarizes the results and shows that the performance of CX2 is much better than the two existing crossover operators with 30 runs.

### 4.1. Benchmark Problems

We perform the proposed crossover operator CX2 along two traditional crossover operators PMX and OX on twelve benchmark instances which are taken from the TSPLIB [28]. In these twelve problems, the ftv33, ftv38, ft53, kro124p, ftv170, rbg323, rbg358, rbg403, and rbg443, are asymmetric and gr21, fri26, and dantzig42 are symmetric TSPs. The experiments are performed 30 times (30 runs) for each instance. The common parameters are selected for GAs, that is, population size, maximum generation, crossover, and mutation probabilities are 150, 500, 0.80, and 0.10, respectively, for less than 100 size instances and results describes in Table 4. Only two changes for more than 100 size instances, that is, population size and maximum generation are 200 and 1000, respectively, and results are described in Table 5. Both tables demonstrate comparison of proposed crossover operator with two existing crossover operators with best, worst, and average results. These results show that the solution quality of the proposed algorithm and with existing crossovers operators are insensitive to the number of runs but number of generations are sensitive, especially in Table 5.

In Table 4, CX2 is performing with other two operators, for instance, gr21 and ftv33, on average basis. Proposed operator gives best average results for instances fri26, ftv38, dantzig42, and ft53. For instance dantzig42, the proposed operator CX2, gives exact optimum value (best known value) sixteen out of thirty times. But for this instance, PMX and OX do not give us an exact value for any run and also we found that the best tour generated with CX2 is 75 and 86 percent shorter than the OX and PMX best tours, respectively. For instance ft53, we found 22 and 26 percent shorter distance than PMX and OX best tours respectively. More interesting aspect about CX2 is that the worst values of dantzig42 and ft53 are much better than others best values.

In Table 5, the results show that all crossover operators work on similar pattern and also found less variations among best, worst, and average values. PMX and OX perform slightly better than CX2 in the instance for rbg323 and for all others instances; the performance of CX2 falls between other two operators except ftv170. For instance, for ftv170, CX2 performs much better than other two with 108 and 137 percent shorter distance of best tours values from PMX and OX, respectively.

Finally, the overall results summarize that the proposed approach CX2 performs better on some aspects similar to existing PMX and OX crossover operators.

## 5. Conclusion

Various crossover operators have been presented for TSP with different applications by using GAs. The PMX and OX along with proposed crossover operator CX2 are mainly focused in this article. At first, we apply these three operators on a manual experiment and found that CX2 performs better than PMX and OX crossovers. Also, for a global performance, we take twelve benchmark instances from the TSPLIB (traveling salesman problem library). We apply all three crossover operators on these benchmark problems. We observe that the proposed operator works over 20, 70, and 100 percent for ft53, dantzig42, and ftv170 problems, respectively, compared to the other two operators. We see that, for large number of instances, the proposed operator CX2 is performing much better than PMX and OX operators. We suggest that CX2 may be a good candidate to get accurate results or may be a fast convergent. Moreover, researchers will be more confident to use it for comparisons.

## Figures and Tables

**Algorithm 1:**
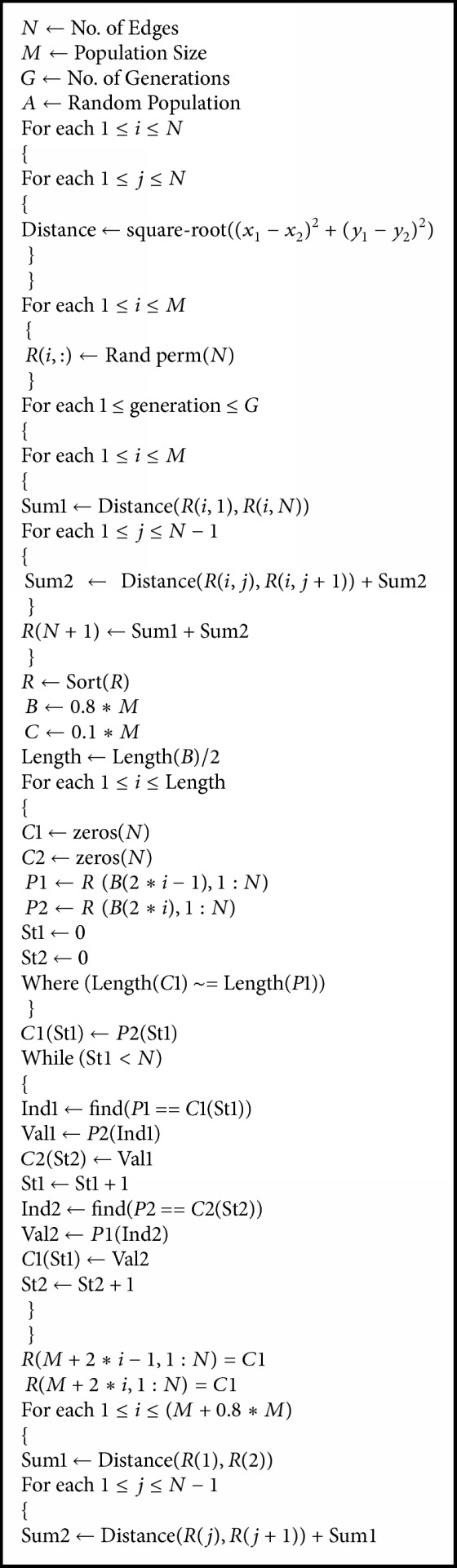
Pseudo-code of proposed algorithm CX2.

**Table 1:** Summary of crossover operators for TSP.

Representation	Crossover operators	Proposed year
Binary	Classical + repair operator	1991

Path	Partially mapped crossover	1985
	Order crossover	1985
	Cycle crossover	1987
	Heuristic crossover	1987
	Order based crossover	1991
	Position based crossover	1991

Adjacency	Alternative edge crossover	1985
	Heuristic crossover 1	1985
	Heuristic crossover 2	1989
	Heuristic crossover 3	1987

Ordinal	Classical operators	1985

Matrix	Intersection crossover	1987
	Union crossover	1987

**Table 2:** Transition distance between cities.

City	1	2	3	4	5	6	7
1	0	34	36	37	31	33	35
2	—	0	29	23	22	25	24
3	—	—	0	17	12	18	17
4	—	—	—	0	32	30	29
5	—	—	—	—	0	26	24
6	—	—	—	—	—	0	19
7	—	—	—	—	—	—	0

**Table 3:** Comparison of three crossover operators (30 runs).

Crossover	Optimum	Average value	Best value	Worst value
PMX	17/30	159.7	159	165
OX	14/30	160.3	159	163
CX2	24/30	159.2	159	162

**Table 4:** Comparison results among three crossover operators.

Instance	*N*	Optimum value	Results	PMX	OX	CX2
			Best	2962	3005	2995
gr21	21	2707	Worst	3322	3693	3576
			Average	3127	3208	3145

			Best	1056	1051	1099
fri26	26	937	Worst	1294	1323	1278
			Average	1133	1158	1128

			Best	1708	1804	1811
ftv33	34	1286	Worst	2399	2366	2322
			Average	2012	2098	2083

			Best	2345	2371	2252
ftv38	39	1530	Worst	2726	2913	2718
			Average	2578	2617	2560

			Best	1298	1222	0699
dantzig42	42	699	Worst	1606	1562	0920
			Average	1425	1301	0802

			Best	13445	13826	10987
ft53	53	6905	Worst	16947	16279	13055
			Average	14949	14724	12243

Generation = 500.

**Table 5:** Comparison results among three crossover operators.

Instance	*N*	Optimum value	Results	PMX	OX	CX2
			Best	090231	097122	092450
kro124p	100	36230	Worst	118386	122497	121513
			Average	100335	103457	101229

			Best	13346	15202	6421
ftv170	171	2755	Worst	19314	19708	8416
			Average	16775	17569	7019

			Best	4123	3998	4212
rbg323	323	1326	Worst	5147	5385	5342
			Average	4434	4602	4654

			Best	5380	5630	5404
rbg358	358	1163	Worst	5915	5948	6004
			Average	5532	5830	5622

			Best	6231	6196	6257
rbg403	403	2465	Worst	6653	6629	6671
			Average	6536	6386	6455

			Best	6754	6932	6854
rbg443	443	2720	Worst	7209	7351	7388
			Average	6905	7121	6981

Generation = 1000.
